# Optimal Swimming Speed in Head Currents and Effects on Distance Movement of Winter-Migrating Fish

**DOI:** 10.1371/journal.pone.0002156

**Published:** 2008-05-14

**Authors:** Jakob Brodersen, P. Anders Nilsson, Jeppe Ammitzbøll, Lars-Anders Hansson, Christian Skov, Christer Brönmark

**Affiliations:** 1 Department of Ecology/Limnology, Lund University, Lund, Sweden; 2 DTU Aqua, National Institute of Aquatic Resources, Silkeborg, Denmark; University of Sheffield, United Kingdom

## Abstract

Migration is a commonly described phenomenon in nature that is often caused by spatial and temporal differences in habitat quality. However, as migration requires energy, the timing of migration may depend not only on differences in habitat quality, but also on temporal variation in migration costs. Such variation can, for instance, arise from changes in wind or current velocity for migrating birds and fish, respectively. Whereas behavioural responses of birds to such changing environmental conditions have been relatively well described, this is not the case for fish, although fish migrations are both ecologically and economically important. We here use passive and active telemetry to study how winter migrating roach regulate swimming speed and distance travelled per day in response to variations in head current velocity. Furthermore, we provide theoretical predictions on optimal swimming speeds in head currents and relate these to our empirical results. We show that fish migrate farther on days with low current velocity, but travel at a greater ground speed on days with high current velocity. The latter result agrees with our predictions on optimal swimming speed in head currents, but disagrees with previously reported predictions suggesting that fish ground speed should not change with head current velocity. We suggest that this difference is due to different assumptions on fish swimming energetics. We conclude that fish are able to adjust both swimming speed and timing of swimming activity during migration to changes in head current velocity in order to minimize energy use.

## Introduction

Getting from one place to another usually requires energy. However, individuals can take advantage of changing environmental conditions to minimize this energy expenditure. This is true both for organisms in nature as well as for modern man, where e.g. more economical airplane flights can be obtained by waiting for beneficial wind conditions and/or adjusting flight speed accordingly. Optimal flight speed is here calculated from wind velocity, engine efficiency and fuel costs. Similar considerations for timing and optimal speed of locomotion can be found in nature, but the question is to what extent animals can estimate when and how fast to travel.

Migratory journeys are widespread throughout the animal kingdom [1] and even beyond (e.g. vertical migration of phytoplankton [2]). Animals may migrate between habitats in a diurnal or a seasonal pattern and do it once, a few or several times during a lifetime. Most often migration is connected to habitat-specific properties considering e.g. foraging, reproduction and predation risk. The time to migrate can often be estimated from such considerations, e.g. from cost/benefit estimations of predation risk versus potential growth rate [3]–[6] or from changing reproductive value of different habitats [7]. Animals may also benefit from adjusting the exact time of migration to environmental conditions, e.g. favourable wind for birds or water currents for fish. Numerous studies on birds have shown that decisions on when to depart on a migratory travel are affected by weather conditions, such as precipitation and wind (reviewed by [8]), and, further, marine animals select favourable tidal currents for their migration [9]–[11]. However, selecting the optimal time to migrate in a relatively unpredictable environment should be a difficult task (but see [12]).

In order to minimize energetic costs, it is not only important to be able to control when to migrate, but also to be able to adjust travel speed to environmental variables. As travel speed can be viewed as a behavioural attribute, variations in travel speed can be analyzed from energy optimization theory. Most research on travel speeds during migration have been conducted on birds, where flight speed is a flexible trait [13]. The two main optimization criteria for migrating birds are to minimize the duration of the migration and to minimize the energy spent on the migration [14]. The importance of the respective criteria is determined by the relative importance of food limitation versus early arrival to the destination [15]. Birds are, furthermore, able to adjust their flight speed to minimize energy use per distance travelled [8], [16], [17]. With respect to fish, a substantial amount of work has been conducted on swimming speed and performances. However, most studies have been performed in swimming flumes in the laboratory and less work has been undertaken in natural conditions, e.g. during natural fish migrations (see however [18] for a review on pacific salmon (*Oncorhynchus spp.*) spawning migration). Early theoretical work predicted that fish swimming against a current should maintain constant ground speed at all current velocities in order to minimize energy use per ground distance [19]. This prediction is still used for estimating optimal ground speed in currents (e.g. [20]). The prediction rests on the assumption that energy expenditure is an exponential function of swimming speed relative to the water, but developments of swimming respirometry have shown that the relationship between swimming speed and energy expenditure may sometimes be better described by a power function [21]. The exponential and power functions that describe how swimming speed affects energy use for fish pose some fundamental differences for the speed-energy relationship. The exponential relationship describes an only modest increase in energy use per increased swimming speed at lower speeds, but a rapid change towards higher energy use per speed increment at higher swimming speeds. The power function, in contrast, describes a more gradual increase in energy use with increasing swimming speed, and does hence not predict any threshold changes in the relationship. But which function that should be considered as generally applicable is still under debate. Still, hitherto no analytical solution to optimal swimming speed in head currents under the assumption of the power function has been provided and numerical solutions have only been provided in few cases (e.g. [22]). In this paper we apply the power function to theoretical predictions, both analytical and numerical, of optimal ground speeds in head currents. We then compare the theoretical results with empirical data from winter migrating cyprinids to evaluate which function, the power or the exponential, best predicts the natural adaptation of swimming speed to variations in head current velocity.

In many systems cyprinids migrate from lakes to streams during winter [23]–[26] and so also in South Swedish Lake Krankesjön, where a large proportion of the roach (*Rutilus rutilus*) population migrates to an inlet stream during winter [25], [26]. This winter migration of roach can be seen as a behavioural strategy to trade off growth for predation avoidance [6], [27], but the migration is also connected with an energy cost. The energy cost of swimming can constitute a high percentage of an individual fish’s total energy budget [28], especially at low temperatures where standard metabolism is low and little energy is used for food consumption. It should therefore be expected that roach would adopt a migration strategy to minimize energy costs and hence time movement according to changing environmental conditions.

In this study we investigate the swimming patterns of migrating fish and relate these to energetic considerations. More specifically, we ask if and how migrating roach adjust their swimming speed and timing of movement to changing head current velocities. To investigate this we use passive telemetry for swimming speed and active telemetry for distance movement estimations and evaluate the results in view of theoretical predictions.

## Results

### Theoretical predictions of optimal swimming speeds in head currents

Energy use for fish during swimming can be described by a power function
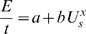
(eqn 1)where *E* is energy expenditure, *t* is time, *U_s_* is swimming speed relative to water and *a*, *b*, and *x* are constants [29]. The constant *a* can be viewed as the standard metabolic rate, whereas the constant *b* is a scaling constant describing the rate at which energy use increases with increasing swimming speed and the constant *x*, often called the speed exponent, describes the curvilinearity of the relationship between swimming speed and energy use. The constants *b* and *x* are related to the body drag and muscle efficiencies [30]. From this follows that the energy used per unit distance, relative to the water, is
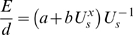
(eqn 2)where *d* is the distance [29]. The optimal swimming speed in stagnant water, often described as cost of transport, can be calculated by letting the first derivative of eqn 2 equal zero and solving for *U_s_*. When swimming in head currents, assuming that *U_s_* = *U_g_*+*U_c_* , where *U_g_* is ground speed and *U_c_* is head current velocity, energy use per distance over ground (*d_g_*) can be calculated as
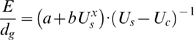
(eqn 3)


To solve 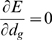 for optimal swimming speed as a function of head current velocity, there are two possibilities. The equation can be solved analytically or numerically. Analytical considerations are provided in Appendix S1, and exact formulae for optimal swimming speed (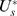) can for mathematical reasons be provided only in the cases where *x* = 2 or *x* = 3:
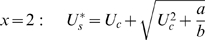
(eqn 4)

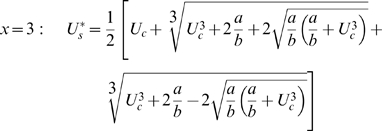
(eqn 5)


**Figure 1:**
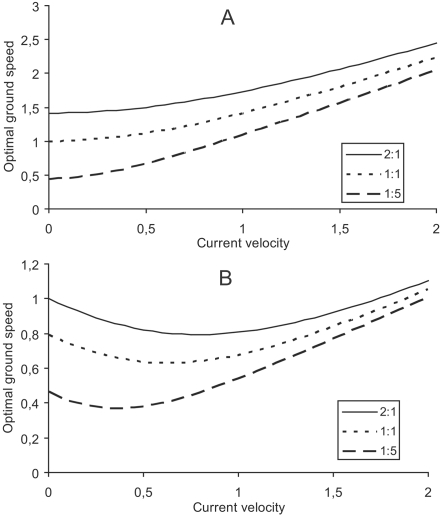
Theoretical predictions of the relationship between current velocity and optimal ground speed for fish swimming against currents. Illustrations are shown for *x* = 2 (A) and *x* = 3 (B) (for equations, see text), and for different *a*∶*b* ratios (2∶1; 1∶1 and 1∶5).

These two cases are however of great interest, since the speed exponent *x* for fish usually lies close to or between these two values (e.g. [21], [30], [31]). For *x* = 2, optimal swimming speed is monotonically increasing with increasing head current velocities and approaches the asymptote 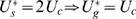 as *U_c_*→∞, where 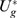 is optimal ground speed (Fig. 1A). For *x* = 3, optimal swimming speed is a non-monotonic function of head current velocity. After an initial decrease it increases and approaches the asymptote 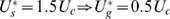 as *U_c_*→∞ (Fig. 1B). Eqn 4 and eqn 5 show that the shape of the curve for a given value of *x* is determined only by the ratio between *a* and *b*, as illustrated in Fig 1. From these theoretical illustrations, we predict that optimal ground speed is not constant for varying head current velocities, but rather increases at a rate depending on the value of *x*.

**Figure 2:**
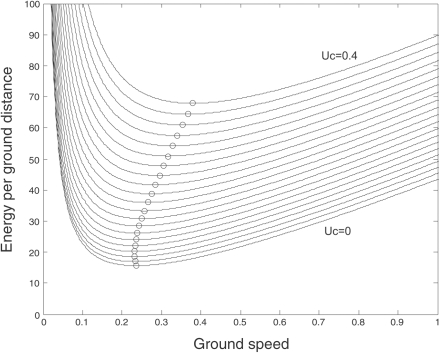
Theoretical predictions on swimming performance of roach (17.0 cm; 55.5 g) swimming at 4.9°C. Energy use as a function of ground speed at different head current velocities in steps of 0.02 ms^−1^ (U_c = _0 for lowest curve; U_c = _0.4 for highest curve). Circles indicate optimal ground speed at a given current velocity.

The above calculations are general for all fish, where the relationship between swimming speed and energy use is described by a power function. Unfortunately, analytical solutions are only available in the cases where *x = *2 or *x = *3. However, when the species, size and temperature-specific constants *a*, *b* and *x* are available, the optimal swimming speed in currents can be found numerically. This, however, requires information on species specific parameter values based on fish size and temperature. For predictions of optimal swimming speed against the water flow for roach, we used the average values for fish length and weight and water temperature found in a passive telemetry study (see below). The constant *a* = 2.25 (assumed to be weight and temperature dependent) refers to standard metabolic rate and is derived from Hölker [32]. Constant *b* = 36.2 (assumed to be length, but not temperature dependent) is derived from Ohlberger et al. [33] and constant *x* = 2.23 (assumed to be independent of temperature and size) as given by Ohlberger et al. [33]. By entering these parameter values in eqn 3, we can numerically analyze the relationship between energy expenditure, ground speed and head current velocity for roach. This analysis shows that at any given head current velocity, a minimum energy expenditure per ground distance can be found, which indicates the optimal ground speed for that particular current velocity (Fig. 2). Furthermore, energy use for keeping a certain ground speed increases with current velocity and optimal ground speed changes with increasing current velocity (Fig. 2). As shown, optimal ground speed is, after a slight initial decrease at very low current velocities, predicted to increase with increasing current velocity. Thus, from a power function, it is predicted that ground speed of winter migrating roach will not be constant at varying head current velocities, as it is predicted from an exponential function, but generally increase with increasing head current velocities at higher current velocities.

### Migration speed (Passive PIT-tag telemetry)

**Figure 3:**
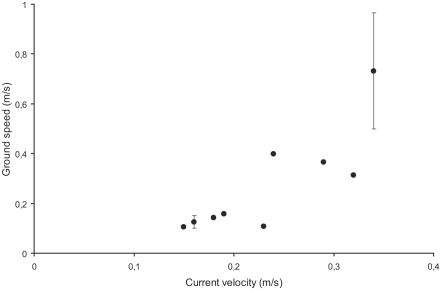
Ground-speed measures of roach moving upstream at different current velocities. Error bars indicate SD on days with more than one upstream migration.

During the study period 25 tagged roach were in contact with the antennae during 35 upstream passages. However, only 14 upstream passages carried out by 10 fish on 9 different dates lasted less than one minute, which was the selection criterion for analysis. Average length and weight (mean±SD) of these fish were 17.0±2.8 cm and 55.5±3.0 g, respectively, and average water temperature for days of passages was 4.9±1.1°C. Ground speed of upstream migrating fish ranged between 0.10 and 0.73 ms^−1^ and was positively related to current velocity (r^2^ = 0.681; *p = *0.006; n = 9; Fig. 3), but not to water temperature or date.

### Spatial movement and habitat use (Active radio telemetry)

**Figure 4:**
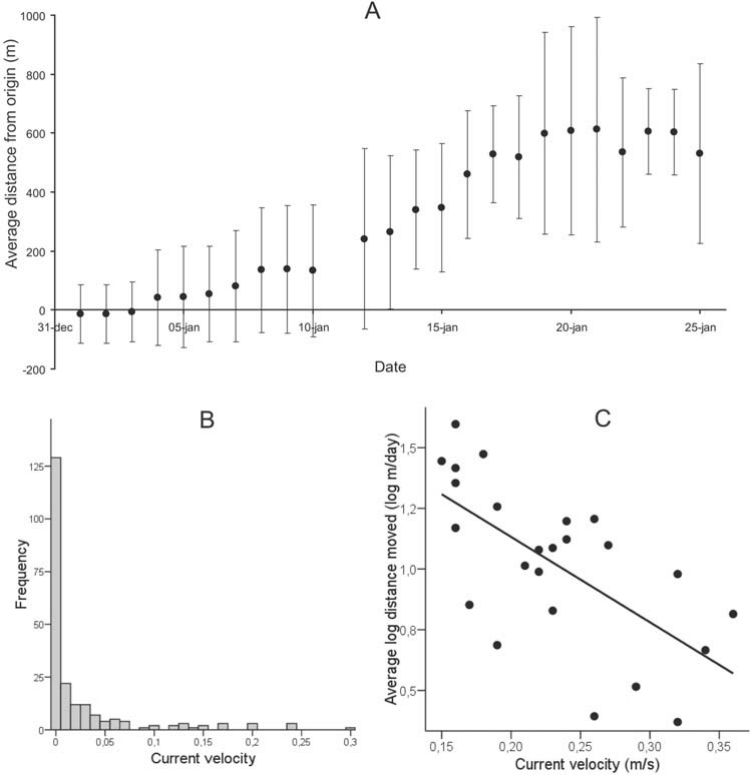
Behaviour of roach during migration. (A) Net upstream distance from first recorded position during the investigation. Error bars indicate SD. (B) Frequency distribution of current velocities at the positions of individual roach in the stream. (C) Average movement undertaken per day by roach as a function of water velocity. Note logarithmic scale on Y-axis.

During the study period the majority of the tagged roach moved upstream from the area around the release location to a side channel of the Silvåkra Stream. Upstream distance from the release site was positively related to time after tagging (r^2^ = 0.946; *p*<0.001; n = 24; Fig. 4A), but not to temperature or water velocity. This shows that the fish had a net upstream movement during the study period, i.e. the study period can be regarded as an active migration period. On the majority of the tracking events, tagged roach were found in habitats with low current velocity (Fig. 4B), most often in vegetation in the beginning of the study period and in slow flowing side channels towards the end. Average distance moved per day was negatively related to the current velocity in the main stream (r^2^ = 0.424; *p*<0.001; n = 24; Fig. 4C) but not to date or temperature.

## Discussion

In our theoretical analysis, a general conclusion is that optimal ground speeds for fish swimming against currents would increase with increasing current velocity. This contradicts previous predictions that fish should have similar optimal ground speed at any current velocity [19]. The difference in these predictions is due to different assumptions on the relationship between swimming speed and energy use in fish, i.e. we assume a power function, as suggested by some authors [e.g. 21], [29], whereas Trump and Leggett [19] assumed an exponential function. Hinch and Rand [22] predicted, from a power function, that optimal ground speed of adult sockeye salmon (*Oncorhynchus nerka*) during spawning migration, would decrease with increasing current velocities, which may seem to contradict our general prediction. However, Hinch and Rand [22] only made predictions for current velocities up to 0.6 ms^−1^. In comparison, we predicted that optimal ground speed of roach should initially drop with increasing current velocities only up to 0.06 ms^−1^, but since adult salmon in warm water have a substantially higher standard metabolic rate than winter migrating roach and thereby a higher ratio between *a* and *b* (eqns 1–4), this difference is expected in accordance with the illustration in Fig 1B. Especially temperature and swimming efficiency are important for the values of *a*, *b* and *x*. Since standard metabolic rate (*a*) increases with temperature, and the speed exponent (*x*) is smaller among efficient swimmers, it is expected that efficient swimmers and fish at high temperatures have relatively high optimal ground speeds in head currents as compared to less efficient swimmers and fish at low temperatures.

Our empirical results from the passive telemetry study corroborated our theoretical predictions derived from a power function, i.e. that ground speed of upstream-migrating fish should increase with increasing current velocity. This suggests that roach are able to adapt their swimming speed to changing head current velocities and that they do so in a way that suggests that their energy use as a function of swimming speed follows a power function and not an exponential function. Most research on fish swimming is carried out under laboratory conditions and studies on adaptive swimming speed in natural environments are few. However, Peake and Farrell [34] found that smallmouth bass (*Micropterus dolomieu*) under semi-natural conditions had higher ground speed at higher water velocities, which is in line with our results.

That fish should swim with higher ground speed when water velocity is high would intuitively lead to the prediction that fish would migrate farther on days with high current velocities. However, performing longer migrations at high water velocities would require more energy than at low water velocities. If refuges from currents are available, for instance in vegetation, fish can instead spend more time in such current refuges and chose to migrate when current velocity is lower. Cyprinids select low velocity habitats in rivers, which can be seen as a strategy to minimize energy expenditure [35], [36]. In accordance with this, we found that fish migrated farther on days with low current velocity, suggesting that roach are able to adapt both swimming speed and timing of migration to changing head current velocities. Similarly, migrating plaice (*Pleuronectes platessa*) control their vertical placement in the tidal current [10], [37], which can be explained by an attempt to maximize the ‘distance covered/energy expenditure’-ratio [38]. However, in the case of plaice, movement is connected to a fixed cycle in water velocity governed by tidal movements of water. In our study, water velocity was related to precipitation and hence not connected to a diurnal cycle. This suggests that fish are able to adjust their movement to both fixed and unpredictable changes in water velocity. That the fish also move during high current velocities could be viewed as exploratory behaviour, as has been seen in migratory flights in birds [8]. Animals have been described to be either time or energy selected in their migration strategies [15]. Our study suggests that roach can be regarded as energy selected, since they utilize current refuges in vegetation awaiting favourable conditions to appear and then migrate when water velocity is low.

Knowledge on fish optimal swimming speed, as provided here, is important for prediction and understanding of routes and timing of fish migration in e.g. anadromous salmon migrations [22], [39], [40], but also in order to understand the general ecology of migrating animals. Furthermore, energy saving migration strategies should be taken into consideration in the construction of fish ways, where resting pools have been suggested to be of importance, since fish after passage of velocity barriers need to recover from burst swimming [20]. Our results suggest that velocity refuges are also of importance for energy-selected migrations where currents are variable, since fish take shelter while waiting for more beneficial current velocities.

## Materials and Methods

### Study site

The study was carried out in the Silvåkra Stream in southern Sweden (55°41’N, 13°29’E), an inlet stream to Lake Krankesjön. The main channel is rich in emergent and submerged vegetation during summer, but towards the end of autumn vegetation decays and mainly emergent vegetation (*Typha sp.*) remains along the bank, where it also decays during winter. Current velocity during the study period ranged between 0.15 and 0.35 ms^−1^, depending on precipitation.

### Migration speed (Passive PIT-tag telemetry)

A total of 1408 roach were individually tagged with passive integrated transponder (PIT) tags [Texas Instruments, RI-TRP-RRHP, half duplex, 134 kHz, 23.1 mm long, 3.85 mm diameter, 0.6 g (air)] in Lake Krankesjön between September 22^nd^ 2003 and December 4^th^ 2004. For further details on tagging procedure see Skov et al. [41]. Two loop-shaped active PIT-antennae, with recording frequency of 5 energize/receive cycle per second, were placed 4.4 m apart along the stream stretch in the Silvåkra Stream 600 meters from the outlet into Lake Krankesjön. The antennae were connected via a multiplexer reader (Texas Instruments) to a single data logger (FlinkaFiskar). As each contact between a tagged roach and an antenna provided data of specimen ID and time of contact, we were able to calculate the ground speed of the roach migrating through the antenna loops. For estimation of length and weight of the fish at the time of passage for fish tagged during the season, we used length-age-weight regressions from survey fishing in the lake during autumn 2005. Current velocities were measured daily with a flow meter (µP-flowtherm, Höntzsch Instruments) in the centre of the stream by the downstream antenna. Since accurate measures of current velocities in the swimming paths of individual fish are extremely difficult, if not impossible, to obtain, it is unreasonable to compare exact values from theoretical calculations with field data on individuals. However, the method is still viable for testing whether general patterns of theoretical predictions hold under field conditions among fish individuals, i.e. in the present study, whether ground speed increases with increasing head current velocity.

### Spatial movement and habitat use (Active radio telemetry)

Tagging of fish took place on the 29^th^ of December 2004. Ten roach were caught by electrofishing in the vegetation in the stream approximately 400 meters upstream the outlet in the lake. The fish ranged between 184 and 244 mm (208±19.6 mm; mean±SD) total length and 67.7 and 164 g in weight (102±33.9 g). The fish were anaesthetized with benzocaine, weighed and measured and each supplied with a small radio transmitter [Advanced Telemetry Systems; model F1520 (length: 19 mm, depth: 8 mm, weight: 1.3 g)] surgically implanted into the stomach cavity (procedure as described by Jepsen and Berg [23]). After tagging, fish were held in an enclosure in the stream for 20 hours before being released in the stream.

After release, the radio-tagged fish were tracked on all, but one (January 11^th^), days between December 31^st^ 2004 and January 28^th^ 2005. On each tracking event, the geographic position of the fish was determined with GPS (Multinavigator, SILVA™). Furthermore, habitat type (e.g. open water, vegetation), current velocity and water temperature at the location of each fish was measured. Each day, current velocity was also measured in the main stream at the place where fish were originally released. Two fish were lost during the investigation period, on the 6^th^ and the 15^th^ day of tracking, respectively. Data obtained from these fish before they were lost was included in the analysis. One transmitter, however, did not move during the study period. Disturbance in the area, where we located the signal, did not cause movement, and thus the fish was assumed to have lost the transmitter or died and was not included in the analysis.

### Ethical considerations

Great care was taken during the tagging procedure to minimize detrimental effects on the fish. PIT-tagging of cyprinids has been shown to have no effect on fish condition or survival [41]. Implantation of radio-transmitters into the body cavity of fish requires more surgery than tagging with PIT-tags. It has, however, been concluded that the method has little effect on fish, as long as the implantation is carried out during temperatures less than 15°C [42], which was the case in the present experiment. The study complies with the current laws in Sweden; ethical concerns on care and use of experimental animals were followed under permission (M14-04) from the Malmö/Lund Ethical Committee.

### Data analysis

It can not be excluded that fish moved in schools and therefore data points from individual fish from each date should not be treated as independent. We thus for each day used average values of all fish both for analysis of daily movement (active telemetry) and swimming speed (passive telemetry). For swimming speed we only used data from fish that took one minute or less to swim from one antenna to the other in order to reduce the chance that fish took cover in vegetation between the antennae during a passage. The a priori choice of selection criteria may seem arbitrary. However, from Fig. 2 it is evident that the lowest expected ground speed is above 0.2 ms^−1^, which corresponds to an expected passage time of less than 22 seconds. Thus, in order to include also slow swimming individuals, we set the maximum passage time to one minute. Allowing longer time for passage would increase the chance of including fish that are taking cover during their passage.

We used multiple regressions with stepwise backward selection (removal criteria: *p*>0.1), for swimming speed (data from passive telemetry) and distance from origin and log daily migratory distance (data from active telemetry) as dependent variables, and date, temperature and water velocity as independent variables. In the case of swimming speed we used linear functions as the main purpose of the test was not to describe the exact function, but rather to test for a positive slope according to our theoretical predictions.

## Supporting Information

Appendix S1 Mathematical calculations and considerations on optimal swimming speed in head currents under the assumption that energy use is a power function of swimming speed
(0.17 MB DOC)
