# Supplementary material for: Optimal Swimming Speed in Head Currents and Effects on Distance Movement of Winter-Migrating Fish
Source: PLoS One. 2008 May 14;3(5):e2156. doi: 10.1371/journal.pone.0002156 (PMC2359855; doi:10.1371/journal.pone.0002156)
Supplement: Appendix S1 — Mathematical calculations and considerations on optimal swimming speed in head currents under the assumption that energy use is a power function of swimming speed (0.17 MB DOC) [file pone.0002156.s001.doc]

**Appendix 1**

**The mathematical problem**

Consider a fish swimming upriver against the current. Insert a coordinate axis along the river with positive direction against the current. Assume that the water in the river flows with a constant velocity *v_c_* > 0. Consider a water particle which at time *t* = 0 is at the origin of the coordinate axis. The position[[1]](#footnote-2) of the particle as a function of time is then given by *s_c_*(*t*) = -*tv_c_*. Let *s_g_* be the positionError: Reference source not found of the fish, and let *s* = *s_g_* – *s_c_* be the distance of the fish to the water particle. Assume that the fish is swimming upriver at all times, i.e

$$v_{g}=\frac{ds_{g}}{dt}=\frac{ds}{dt}+\frac{ds_{c}}{dt}=v-v_{c}>0.$$

Here *v_g_* and *v* are functions of *t*. Thus, we assume that $v_{g}\left(t\right)-v_{c}>0$ for all t.

We assume that the amount of energy that the fish is using per time unit is described by a power function given by

where, $\tilde{a}=\frac{a}{bv_{c}^{x}}\in\mathbf{R}$, $\tilde{b}=bv_{c}^{x}\in\mathbf{R}$and $x\in\mathbf{R}$ are constants which satisfy the inequalities and $\tilde{b}x\geq0$, and where $f:\left[0,\infty\right[\to\mathbf{R}$is defined by

Our assumptions on $\tilde{a},\tilde{b}$and *x* implies that

so that the fish always provides a positive effect, which is increasing with the speed of the fish.

The amount of energy used per unit of distance travelled upriver is given by

$$\frac{dE}{ds_{g}}=\frac{\frac{dE}{dt}}{\frac{ds_{g}}{dt}}=\frac{f\left(\frac{v}{v_{c}}-1\right)}{v-v_{c}}=\frac{1}{v_{c}}\cdot\frac{f\left(\frac{v}{v_{c}}-1\right)}{\frac{v}{v_{c}}-1}$$

We seek to determine a constant value *v* > *v_c_* of the function *v* which minimizes this value. Put $u=\frac{v}{v_{c}}-1$. Our task is then to minimize

$u\mapsto\frac{f\left(u\right)}{u}=h\left(u\right)$ for $u\in\mathbf{R}$

**Existence and uniqueness of a solution**

**Case** $\tilde{b}x=0$. In this case, is a positive constant, and *h* is minimized by choosing *u* as large as possible.

**Case** $\tilde{b}x>0$. Since $f'>0$in this case, we find that

where $F:\left[0,\infty\right[\to\mathbf{R}$ is defined by

We see that *F*(0) < 0, and that , and thereby

Since

and both $\left(1+u\right)^{x}-1$and have the same sign as *x* (except for *u* = 0, where $\left(1+u\right)^{x}-1=0$), we find that

(1) $\mathrm{sgn}\left(F'\right)()=\mathrm{sgn}\left(x-1\right)\mathrm{sgn}\left(x\right)\mathrm{sgn}\left(x\right)=\mathrm{sgn}\left(x-1\right).$

We now subdivide the case under consideration in two.

**Case** $\tilde{b}x>0,x\leq1$. Since *F*(0) < 0 and $F'\leq0$(according to (1)), *F* is negative. *h* is thereby strictly decreasing. Therefore, *h* is minimized by choosing *u* as large as possible.

**Case** $\tilde{b}x>0$, *x* > 1. We have *F*(0) < 0 and $F'>0$(according to (1)). Since $F'\left(u\right)\to\frac{x-1}{x}>0$for $u\to\infty$, $F\left(u\right)\to\infty$for $u\to\infty$. Thus, *F* is strictly increasing, and there exist a unique $u\in\mathbf{R}_{+}$ that minimizes h.

**Properties of the solution**

In the remaining part of the text we consider the case $\tilde{b}x>0$, *x* > 1, in other words the case where $\tilde{a}>-1$, $\tilde{b}>0$ and *x* > 1. In this case we have found that there exists a unique *u* minimizing $h:\mathbf{R}_{+}\to\mathbf{R}_{+}$. This *u* is characterized by the following equivalent conditions:

$$\left(x-1\right)\left(1+u\right)^{x}-x\left(1+u\right)^{x-1}-\tilde{a}=0$$

$$g\left(u,x\right)=\tilde{a},$$

where $g:\mathbf{R}_{+}\times\left]1,\infty\right[\to\mathbf{R}$ is defined by

$$g\left(u,x\right)=\left(x-1\right)\left(1+u\right)^{x}-x\left(1+u\right)^{x-1}.$$

The last condition shows that the minimizing *u* depends only on $\tilde{a}$ and *x*, and not on $\tilde{b}$. The minimizing *u* can thus be regarded as a function of $\tilde{a}$ and *x*. This function, , is implicitly defined by

(2) for all $\tilde{a}>-1$, *x* > 1.

We find that

$$\begin{matrix} \frac{\partial g}{\partial u}\left(u,x\right)=x\left(x-1\right)u\left(1+u\right)^{x-2} \\ \frac{\partial g}{\partial x}\left(u,x\right)=\left(1+u\right)^{x-1}\left(u+\left(\left(x-1\right)u-1\right)\log\left(1+u\right)\right). \end{matrix}$$

Since $\frac{\partial g}{\partial u}>0$, the Implicit Function Theorem shows that $u:\left]-1,\infty\right[\times\left]1,\infty\right[\to\mathbf{R}_{+}$ is differentiable of class $C^{\infty}$. Thus, the optimal speed of the fish is a ‘nice’ function of the parameters $\tilde{a}$ and *x*, even though we are not able to express this function explicitly using the elementary functions.

For certain values of $\tilde{a}$ and *x* it is however possible to give explicit formulae for . The theorem below gives three such formulae

THEOREM

$$u\left(0,x\right)=\frac{1}{x-1}$$

*when* $\tilde{a}\geq0$.

PROOF. The first equation follows easily from (2). The other two are based on the observation that *g*(*u*,*x*) is a second or third degree polynomial in (1 + *u*) when *x* equals 2 or 3 respectively. Hence $g\left(u,x\right)=\tilde{a}$can be solved explicitly for *u* in these cases. In the case *x* = 3 use Cardano’s formula.

Differentiation of equation (2) with respect to $\tilde{a}$ gives that

and thereby

Thus *u* is strictly increasing with $\tilde{a}$ (the higher the $\tilde{a}$, the faster the fish should swim).

Differentiation of equation (2) with respect to *x* gives that

and thereby

To investigate the sign of this, we notice that

The formula for $\frac{\partial g}{\partial x}$now shows that

when $\tilde{a}\geq0$(and *x* > 1).

Thus, when $\tilde{a}\geq0$, *u* is strictly decreasing with *x* (the larger the *x*, the slower the fish should swim).

1. the signed distance to the origin of the coordinate axis. [↑](#footnote-ref-2)
